# Unusual Presentation of a Rapidly Progressive Coronary Artery Pseudoaneurysm after Drug Eluting Stent Placement

**DOI:** 10.7759/cureus.13305

**Published:** 2021-02-12

**Authors:** Sreenivas Reddy, Raghavendra Rao K, Tek Singh Mahant, Sandeep Goel, Sreedhara B Cheluvashetty

**Affiliations:** 1 Department of Cardiology, Government Medical College and Hospital, Chandigarh, IND; 2 Department of Cardiovascular and Thoracic Surgery, Fortis Hospital Mohali, Mohali, IND; 3 Department of Cardiology, Chandigarh Heart Centre, Sangrur, IND; 4 Department of Radiodiagnosis and Imaging, Postgraduate Institute of Medical Education and Research (PGIMER), Chandigarh, IND

**Keywords:** percutaneous coronary intervention, drug eluting stent, coronary artery aneurysm

## Abstract

Infected coronary artery aneurysm (CAA) is a rare complication of percutaneous coronary intervention (PCI) and is associated with high morbidity and mortality. The management of infected CAA is unclear and is based on the clinical and imaging features. We report an interesting case of a giant infected right CAA secondary to Pseudomonas aeruginosa within four weeks of a drug eluting stent (DES) implantation. Chronological analysis of the coronary angiograms and computed tomography coronary angiography revealed rapid progression in the size of the aneurysm from small to a giant CAA over a period of four weeks. Patient remained afebrile throughout the hospital stay without any signs of septicaemia. In view of the rapid progression in size, surgical aneurysmal ligation with distal revascularisation was done with good post-operative recovery. Afebrile presentation of an infected CAA is very rarely reported in the literature as in our case. Early diagnosis using multimodality imaging and immediate surgical intervention are the cornerstone in the management of giant infected CAAs.

## Introduction

The incidence of coronary artery aneurysm (CAA) after coronary intervention ranges from 0.3% to 6.0%, with true aneurysms being lesser in incidence compared to the pseudoaneurysm [1]. In the current era, the drug eluting stent (DES) has replaced the bare metal stent (BMS) in view of their favourable outcomes [2]. Various mechanisms have been postulated as the potential cause of CAA after DES implantation, such as the anti-inflammatory and antiproliferative properties of the drug resulting in delayed healing and endothelization of the injured vessel wall after percutaneous coronary intervention (PCI), vascular hypersensitivity reactions to either the metal, drug or polymer [3] and mechanical factors such as coronary artery dissections, injury to the vessel media caused by large-sized balloons or stents, atherectomy and laser angioplasty [1]. Very rarely, Kawasaki disease and coronary stent infections have been mentioned as the causative factors for CAA [4,5].

Coronary stent infection is an extremely rare and life-threatening complication of PCI which has varied presentations such as CAA, pericardial effusion, abscess, and coronary cameral fistula [6-8]. The majority of coronary stent infections are caused by Staphylococcus aureus (80%) followed by Pseudomonas aeruginosa (20%). Herein, we report an unusual presentation of a rapidly progressive, giant infected coronary artery pseudoaneurysm secondary to Pseudomonas aeruginosa after a DES placement, managed successfully with surgical intervention.

## Case presentation

A 56-year-old hypertensive and diabetic male presented to our tertiary care center with complaints of retrosternal chest discomfort of one-month duration. Physical examination was unremarkable with a pulse rate of 78 beats per minute and blood pressure of 130/80 mm Hg. The past medical history was significant for ST-segment elevation inferior wall myocardial infarction one month back for which he underwent a primary PCI of the right coronary artery (RCA) at a local hospital. Cardiac enzymes creatine kinase (CK-MB) and troponin-T were elevated and the echocardiogram revealed inferior wall hypokinesia with a left ventricular ejection fraction of 50%.

**Figure 1:**
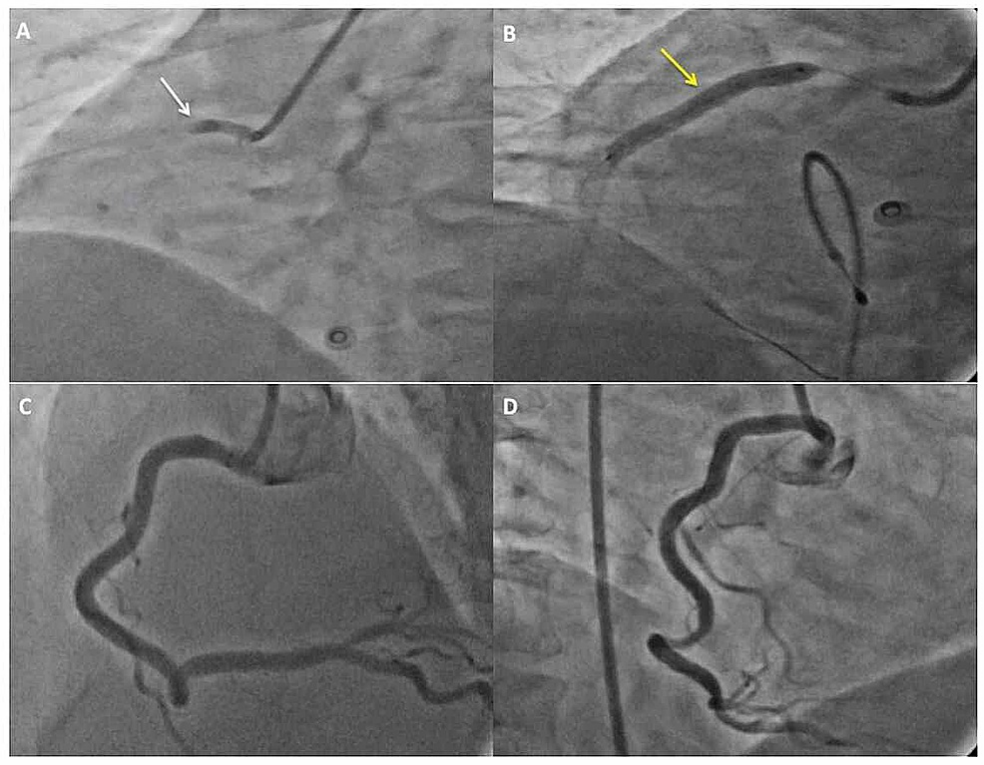
Baseline coronary angiographic view Baseline coronary angiographic views, (A) left anterior oblique (LAO) cranial view showing 100% occluded proximal right coronary artery (RCA) (white arrow); (B) Deployment of stent (yellow arrow) in proximal to mid RCA; (C) Post percutaneous coronary intervention (PCI) LAO cranial view; (D) Post PCI right anterior oblique (RAO) view.

After giving the loading doses of aspirin, clopidogrel, and statins, the patient was taken for coronary angiography which revealed a proximal 100% occlusion of the dominant RCA (Figure 1A). Following administration of therapeutic doses of intravenous heparin titrated according to the activated clotting time (ACT), the patient was taken up for primary PCI of RCA under all aseptic precautions, with the back-up of a temporary pacemaker. RCA was engaged with a Judkins Right 3.5 guiding catheter (6 French) and the lesion was crossed using a 0.014" Asahi Sion Blue guidewire (Asahi Intecc, Japan). The lesion was pre-dilated with the help of a 2.5 x 15 mm semi-compliant Maverick balloon (Boston Scientific, Natick, Massachusetts, USA) at 10 to 14 atm pressures. The lesion was stented successfully using a 4 x 38 mm Promus Element Plus stent (Everolimus-Eluting Platinum Chromium Coronary stent system; Boston Scientific, Natick, Massachusetts, USA) deployed at 10 atm pressure (Figure 1B). The stent was post-dilated using a 4 x 8 mm non compliant Quantum Apex balloon catheter (Boston Scientific, Natick, Massachusetts, USA) at 10 to 14 atm pressures. As the index PCI procedure was done at a referral hospital, the details of the usage of resterilized equipments were not available. The procedure was uneventful with a post-procedure angiography showing distal thrombolysis in myocardial infarction (TIMI) III flow (Figures 1C-1D). The temporary pacemaker was removed after 24 hours and the patient was discharged on the third post-operative day in a stable condition.

**Figure 2:**
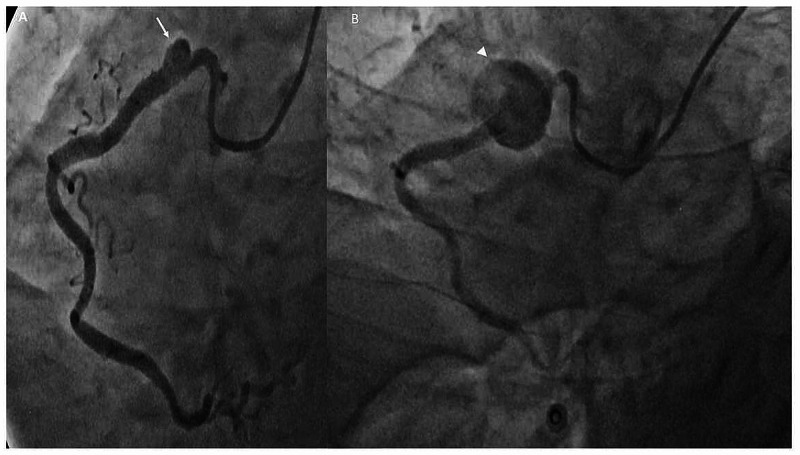
Sequential cine angiograms Sequential cine angiograms (A) left anterior oblique (LAO) caudal view demonstrating a small pseudoaneurysm (white arrow) at the proximal stent edge taken two weeks post percutaneous coronary intervention (PCI); (B) LAO caudal view done four weeks post PCI demonstrating a progression in the size of pseudoaneurysm (white arrow head) at the proximal stent edge.

The patient was readmitted two weeks after the PCI at an outside hospital with complaints of retrosternal chest discomfort and a check angiogram done revealed a small sized RCA aneurysm measuring 5.5 x 3.8 mm at the proximal stent edge (Figure 2A). Patient was kept on conservative management, however, the patient had recurrence of chest pain one week later, for which a repeat coronary angiogram was done at an outside hospital which showed a progression in the size of the right CAA to 15 x 13 mm (Figure 2B).

**Figure 3:**
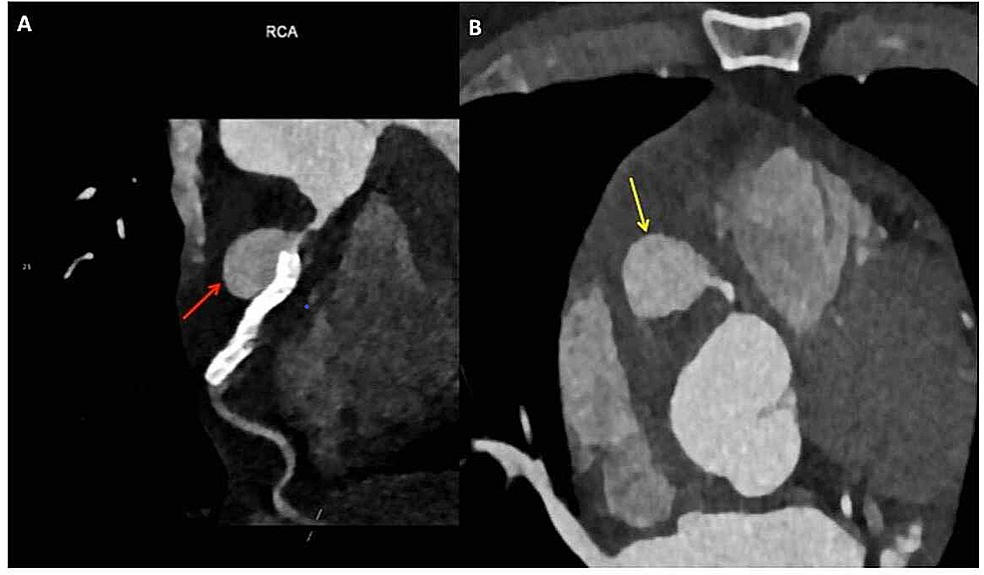
CT coronary angiography (A) CT coronary angiography oblique view of the right coronary artery (RCA) shows the large pseudoaneurysm at the proximal end of the stent arising approximately 12 mm from the ostium (red arrow); (B) CT coronary angiography axial view at the level of aortic sinus shows the pseudoaneurysm along the course of the RCA (yellow arrow).

**Figure 4:**
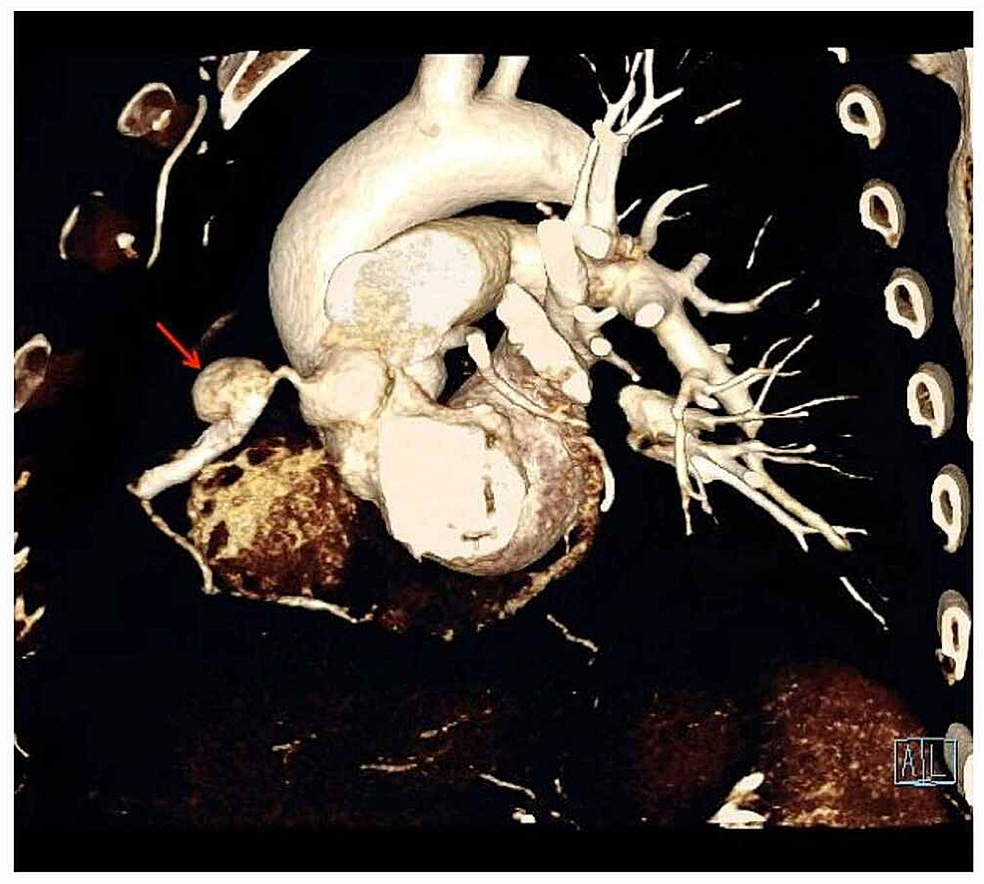
CT coronary angiography: curved volume rendering technique (VRT) image Curved VRT image showing the large pseudoaneurysm at the proximal stent edge (red arrow).

The patient was received at our institute four weeks post-PCI for further management and to delineate the exact anatomical details of the aneurysm; the patient underwent CT coronary angiography which showed a 24 x 17 mm large pseudoaneurysm (Figures 3-4) arising from proximal RCA approximately 12 mm from the ostium along the proximal end of the RCA stent. The flow within the stent distally and the rest of the RCA was maintained. Mild focal ectasia was noted at the distal end of the stent. 

**Figure 5:**
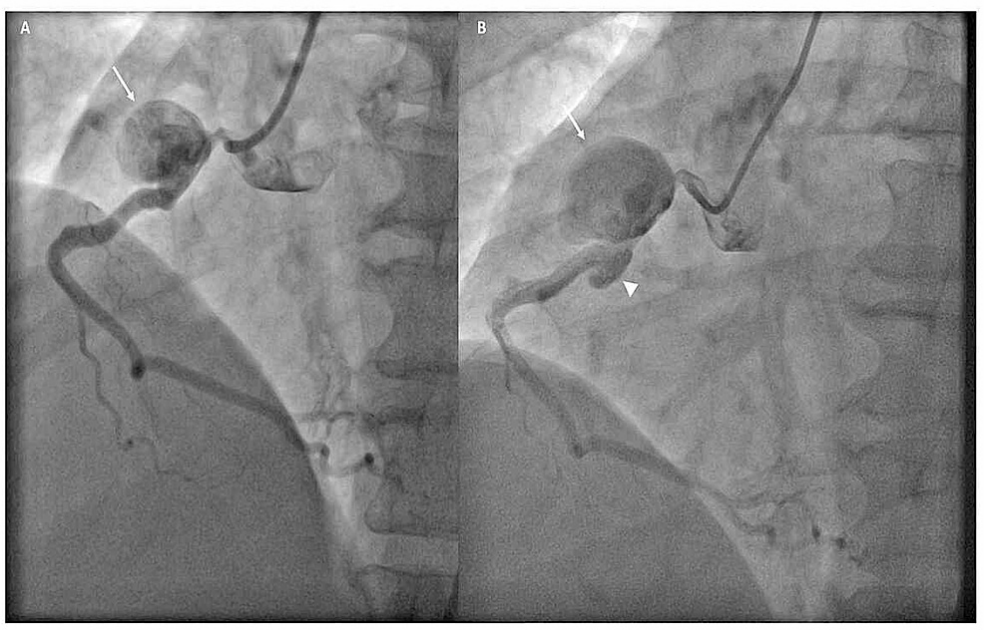
Sequential cine angiograms (A) Cine angiogram in left anterior oblique (LAO) cranial view showing a 22 mm x 15 mm pseudoaneurysm arising at the level of proximal stent edge (white arrow); (B) Cine angiogram in LAO view showing a large 26 mm x 19 mm pseudoaneurysm arising at the level of proximal stent edge (white arrow) with inferior extension (white arrow head).

Coronary angiography done after presentation to our institute showed a large RCA pseudoaneurysm measuring approximately 24 x 17 mm in size (Figure 5A). However, in view of mild increase in chest pain, a check angiogram was done four days later which showed a rapid increase in the size of the aneurysm to 26 x 19 mm with inferior extension and hence, the patient was further planned for surgical resection of the aneurysm and distal revascularization using bypass graft (Figure 5B).

**Figure 6:**
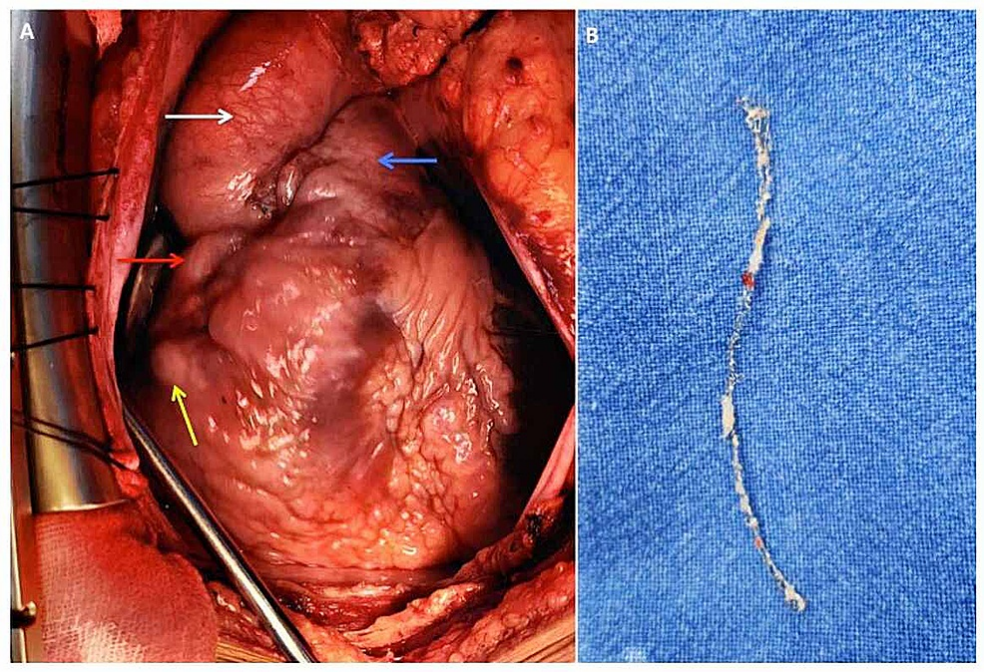
Surgical images Surgical images demonstrating (A) the protruding right coronary artery (RCA) aneurysmal sac containing blood mixed with pus (yellow arrow), right atrium (red arrow), aorta (white arrow), pulmonary artery (blue arrow); (B) extracted RCA stent.

Cardiopulmonary bypass was established and RCA aneurysm was identified and incised to remove the stent in total (Figures 6A-6B).

The aneurysmal sac contained blood mixed with pus which was removed and the cavity was washed with antibiotics. The proximal and distal RCA were ligated and the distal end of the venous graft was anastomosed to the posterior descending artery. Histopathological examination of the aneurysmal tissue showed fibrin with entrapped leucocytes along with RBC’s and the tissue culture grew Pseudomonas aeruginosa. Patient was treated with IV antibiotics according to the culture sensitivity for four weeks and was discharged in a stable condition. Patient had a good postoperative recovery and remained asymptomatic on follow-up.

## Discussion

Infected CAAs are very rare in their occurrence and various hypotheses have been proposed for the pathogenesis of mycotic aneurysms such as micro embolization to the vasa vasorum, pathogen invasion of the arterial wall, or immune complex deposition, resulting in medial injury and destruction [9,10]. It has also been hypothesized that DES might increase the susceptibility to infection because of the antiproliferative and immunosuppressive effects of the drug hampering the local host defense mechanism as well as delays the process of endothelization [5,11]. The overall incidence of bacteremia after invasive nonsurgical cardiologic procedures such as diagnostic angiography, PCI, and electrophysiologic studies within 72 hours was 0.11% [12]. The etiology and factors predisposing to peri-procedural infection is unknown. However, the major risk factors for developing infectious complications after angioplasty are advanced age, repeat percutaneous puncture of the same artery, an indwelling arterial sheath with repeat procedure in the same sheath that remained for several days, and blood oozing around the arterial sheath [8,9,13]. Our patient did not have these risk factors.

The most common presenting symptom of infected CAA is fever and our patient was peculiar as he was afebrile throughout the presentation [8]. Table 1 shows the reported cases of coronary stent infections secondary to Pseudomonas aeruginosa in the literature and all the reported patients had fever as the presenting symptom but our patient was unique as he was afebrile throughout the presentation.

**Table 1:** Reported cases in the literature of coronary intervention related infections secondary to Pseudomonas aeruginosa RCA: Right coronary artery; LAD: Left anterior descending artery; LCX: Left circumflex artery

Author (year)	Age	Artery	Coronary Intervention	Index Procedure to symptom onset duration	Presenting symptom	Vessel pathology	Surgery	Outcome
Timsit JF et al. [14] (1993)	66 years	RCA	Balloon angioplasty	2 days	Fever	RCA to aortic sinus fistula	Surgery	Survived
Leroy O et al. [15] (1996)	49 years	LAD	Bare metal stent	7 days	Fever	Aneurysm	Surgery	Died
Bouchart F et al. [13] (1997)	38 years	LCX	Bare metal stent	6 days	Fever	Pseudoaneurysm	Surgery	Survived
Furtado AD et al. [16] (2011)	62 years	LAD	Drug eluting stent	2 weeks	Fever/chest pain	Pseudoaneurysm	Surgery	Survived
Sangolkar R et al. [7] (2018)	66 years	RCA	Drug eluting stent	12 months	Fever	Coronary cameral fistula	Surgery	Survived
BGK Sudhakar [17] (2018)	49 years	LAD	Drug eluting stent	2 weeks	Fever	Aneurysm	Surgery	Survived
Singh AP et al. [18] (2020)	43 years	LCX	Drug eluting stent	3 months	Fever	Aneurysm	Surgery	Died
Present case	56 years	RCA	Drug eluting stent	1 month	Chest pain	Pseudoaneurysm	Surgery	Survived

The infected CAA can present anytime from day 1 to up to 11 months postintervention, with most cases presenting within the first month as in our case [5,19]. The most common organism associated with coronary artery stent infection is Staphylococcus aureus (80%) followed by Pseudomonas aeruginosa (20%) and our patient had grown pseudomonas aeruginosa from the tissue culture [8,19]. Infected CAAs carry significant morbidity and mortality and complications include thrombosis, distal embolization, rapid expansion, and rupture [9,10]. Our patient was unique as he had a rapid progression in size from a small to a giant aneurysm within one month of the coronary intervention and remained afebrile throughout the hospitalization despite having Pseudomonas aeruginosa infection. Not all cases of infected CAA manifest with fever, hence the nonspecific nature of the symptoms often leads to delay in diagnosis and treatment, requiring a high clinical suspicion [20].

The most common diagnostic modality used to diagnose infected CAA is coronary angiography as in our case, although other investigations such as transthoracic echocardiography, transesophageal echocardiography, CT, and cardiovascular magnetic resonance imaging are also utilized [5,8]. A radiolabelled leucocyte scan or a PET-CT (positron emission tomography-computed tomography) can be utilized as a diagnostic modality to confirm or refute the diagnosis of infected CAA, if conventional imaging fails to identify the source of infection [5,7]. Apart from antibiotic and antiplatelet therapy, the mainstay of treatment in patients with infected CAA remains early surgery as there is a high risk of rupture with significant morbidity and mortality, however, covered stent maybe an alternative treatment option in high risk surgical candidates [8,9,19,20].

## Conclusions

Infected CAA carries significant morbidity and mortality if not treated timely. Majority of patients present with fever within one month of coronary intervention, although rarely afebrile presentations are also seen requiring a high degree of clinical suspicion to prevent complications. This case highlights the importance of considering the differential diagnosis of infected CAA in a patient presenting with chest pain after a recent PCI. Antibiotics and early surgery remain the cornerstones in the management of large infected CAA.
